# Novel start codon variant in the 5’UTR of *LDLR* associated with familial hypercholesterolaemia

**DOI:** 10.1038/s41431-025-01893-y

**Published:** 2025-07-24

**Authors:** Martin Bird, Chris Jyun-Peng Tung, Alan M. Pittman, Elijah R. Behr, Axel Nohturfft, Marta Futema

**Affiliations:** 1 Cardiovascular and Genomics Research Institute, School of Health & Medical Sciences, City St George’s, University of London, London, UK; 2 Neuroscience and Cell Biology Research Institute, School of Health & Medical Sciences, City St George’s, University of London, London, UK; 3 Institute of Cardiovascular Science, Faculty of Population Health, University College London, London, UK

**Keywords:** Gene expression, Dyslipidaemias

## Abstract

Familial hypercholesterolaemia (FH) is a genetic disorder due to pathogenic variants in *LDLR*, *APOB*, and *PCSK9* genes, characterised by elevated low-density lipoprotein cholesterol (LDL-C) concentration and a significantly increased risk of premature coronary heart disease. Annotating whole genome sequencing data of 536 FH patients using the VEP plugin UTRannotator, we identified a novel variant c.−35C > G in the 5’ untranslated region (5’UTR) of *LDLR*, predicted to introduce an upstream translation initiation codon and upstream open reading frame (uORF) that is out of frame with the *LDLR* coding sequence. Using promoter and epitope reporter assays, we demonstrate that the c.−35C > G variant leads to the preferential utilisation of the upstream AUG codon over the wild-type *LDLR* translation start site. We additionally conducted reporter assays for a previously reported variant that introduces a novel AUG codon through a deletion at position −22 of the 5’UTR (c.−22del) and obtained similar results. These findings confirm a novel type of FH-causing *LDLR* variants, leading to a premature start of translation and a truncation, underscoring the need for expanded genetic screening beyond coding regions. Future studies should focus on further characterising 5’UTR variants to better understand their role in FH.

## Introduction

Familial hypercholesterolaemia (FH) is a genetic disorder marked by elevated levels of low-density lipoprotein cholesterol (LDL-C), which increases the risk of developing premature coronary heart disease (CHD) [1]. Clinical diagnosis of possible FH is widely based on the well-established Simon Broome and Dutch Lipid Clinic Network criteria [2]. Where probands meeting the clinical FH criteria were followed up by sequencing, most of the identifiable pathogenic variants have been found in the *LDLR*, *APOB*, and *PCSK9* genes [1, 3–5]. Defects in the proteins encoded by these genes result in impaired clearance of circulating LDL particles, leading to a lifelong burden of increased cholesterol in affected individuals [6]. More recently, a specific variant in the *APOE* gene (p.Leu167del) has also been linked to FH [7, 8]. Additionally, a recessive form of hypercholesterolaemia can arise from homozygous or compound heterozygous pathogenic variants in *LDLRAP1* [9].

Variants in the protein-coding region of the *LDLR* gene make up the great majority ( > 80%) of genetic changes that are known to cause FH [10, 11]. Moreover, FH-associated variants have been identified in the promoter region of *LDLR* that are predicted to inhibit the binding of important transcription factors such as sterol regulatory element-binding proteins (SREBPs), specificity protein 1 (Sp1), cAMP responsive element-binding protein (CREB), and TATA box binding protein. In functional studies, these variants showed 3–72% wild-type promoter activity [12].

Currently, there is limited functional evidence supporting the pathogenicity of 5’UTR variants. To date, only 2 *LDLR* 5’UTR (region c.-86 to c.−1) variants have been functionally studied (c.−13A > G, c.−36T > G), both showing no effect on promoter activity [11–13]. These variants are likely benign because they do not create new upstream start sites (uAUG) or upstream open reading frames (uORF). To our knowledge, only one instance of a uORF creating variant in *LDLR* has been described in a Turkish male proband with FH who was found to be compound heterozygous for a c.−22del variant resulting in an uAUG [14]. However, no functional analyses of this variant have been reported.

The prevalence of FH-causing variants in known FH genes is estimated to be around 1 in 250 individuals (95% CI 1:345–1:192) across diverse populations, including those of South Asian and African ancestry [15, 16]. Early diagnosis and proactive lipid-lowering treatments are crucial for minimising the likelihood of cardiovascular events in this group [17]. Identifying the pathogenic variant in individuals with FH is crucial not only for guiding targeted treatment but also for effective cascade testing in family members, thereby enabling early detection of those at risk. However, in mutational analyses at least half of probands with clinical FH remained without a genetic diagnosis [1, 3–5], which highlights the importance of exploring non-coding regions and other genetic contributors. Here, using whole genome sequencing (WGS) data we apply in silico annotation tools to look specifically for 5’UTR variants, followed by functional testing. In our analyses of promoter activity, we included the previously published variant c.−22del which creates a novel uAUG, to provide novel insights into the regulatory mechanisms that contribute to FH and the role of non-coding variants in the disease.

## Methods

### 100,000 Genomes project familial hypercholesterolaemia cohort

The current study (project ID: RR123) has been approved by the Genomics England Clinical Interpretation Partnership (GeCIP) cardiovascular domain committee [18]. The study cohort comprised 467 probands and 69 affected relatives, classified as having Possible or Definite FH based on the Simon Broome diagnostic criteria, as per National Institute for Health and Care Excellence (NICE) recommendations [2]. Inclusion criteria were LDL-C levels exceeding 4.9 mmol/L and a family history of myocardial infarction or severe hypercholesterolaemia. Participants were recruited through the Rare Diseases: Cardiovascular domain of the 100,000 Genomes Project (100KGP). This study follows our previous bioinformatic analyses of the FH 100KGP whole genomes to provide new knowledge on a variant of uncertain significance (VUS) [19].

### In silico 5’UTR variant annotation

UTR variant annotation utilised ensembl Variant Effect Predictor (VEP) [20] v99 in conjunction with UTRannotator [21]. Variants which were not present or had a minor allele frequency (MAF) ≤ 0.0001 in GnomAD v4.1.0 were retained. The variants that were selected, were further classified to one of the following types of high impact 5’UTR variants: uAUG gain, uAUG loss, upstream stop site (uSTOP) loss, and upstream frameshift (uFrameshift). Variants identified after this filtering process were then searched for in the rest of the 100KGP WGS participants to verify if they were unique to the 100KPG FH cohort.

### Plasmids

Reagents are listed in the Supplementary Methods S1. Plasmids phRL-CMV (expressing *Renilla reniformis* luciferase), pGL4.32 and pGL4.36 were purchased from Promega and plasmid pCMV-βGal from Stratagene. Plasmid pANG32 expresses the Luc2P reporter gene (*Photinus pyralis* luciferase fused to an hPEST protein destabilisation sequence) from a promoter containing five copies of an NF-κB response element and was generated by cutting pGL4.32 with XbaI, followed by religation to remove the hygromycin expression cassette. To generate plasmid pANG183, the HindIII-AdeI fragment of pANG32 was replaced with that of pGL4.36, which removed the TATA box. Plasmid pLDLR-Luc2P was derived from pANG183 by replacing the KpnI-HindIII fragment with a sequence that includes nucleotides −428 to −91 of the human *LDLR* gene (all coordinates are relative to translation start site) [22]. In plasmid pLDLR-UTR-Luc2P and its derivatives the reporter is driven from nucleotides −428 to −1 of the *LDLR* gene with the consensus start site of transcription at -86 (MANE transcript ENST00000558518.6); the constructs were generated by replacing the BmgBI-NcoI fragment of pLDLR-Luc2P with annealed oligonucleotides corresponding to nucleotides −97 to −2 (see Supplemental Methods S2). Plasmid pLDLR-UTR-HiBiT-Luc2P and related constructs differ from pLDLR-UTR-Luc2P by the fusion of a sequence expressing an 11-amino acid HiBiT peptide tag to the N-terminus of Luc2P [23]. These plasmids were constructed by recombining BmgBI and NcoI-digested pLDLR-Luc2P with double-stranded DNA fragments using NEBuilder HiFi DNA Assembly reagent (New England Biolabs, Hitchin, UK). All plasmids were verified by sequencing.

### Cell culture

Medium A refers to DMEM/F12 supplemented with antibiotics (100 units per ml of penicillin and 100 µg per ml of streptomycin sulphate) and 10% FBS (Fetal bovine serum). Medium B refers to DMEM/F12 supplemented with antibiotics, 5% lipoprotein-deficient serum (LPDS), 0.5 µM lovastatin and 50 µM mevalonate. Human alveolar adenocarcinoma A549 cells (ATCC CCL−185) were grown at 37 ˚C in an atmosphere of 5% CO_2_ in medium A.

### Plasmid transfection and enzyme assays

A549 cells were transfected using TurboFect™ reagent according to the manufacturer’s instructions (Thermo Fisher Scientific, MA, USA) at a ratio of 3 µl Turbofect per µg DNA. To measure the activities of *P. pyralis* and *R. reniformis* luciferases, cells in 96-well plates were lysed and analysed with Dual-Glo Luciferase Assay reagents (Promega) as described [24]. Expression of the HiBiT tag was detected based on its binding to the LgBit complementation partner, which reconstitutes a NanoLuc luciferase activity that was measured using the Nano-Glo® HiBiT Lytic Detection System (Promega). To measure the activity of bacterial β-galactosidase expressed from the pCMV- βGal plasmid, A549 cells in 96-well plates were lysed with 50 µl lysis buffer and incubated on ice for 30 min. For β-Gal assays, 20 µl of lysate were mixed with 30 µl of 5 mM ONPG in a black-walled clear-bottom microplate and incubated at 37 ˚C for 1–3 h. Luminescence and 405-nm absorbance readings were performed using a Promega GloMax® Discovery microplate reader. Linearity of the activity signals was verified by serial dilutions.

### Statistics

Normal distribution of relative luciferase expression data was assessed in R using the Shapiro test. To assess differences between data groups, an unpaired Student’s t test was performed where each dataset was normally distributed; otherwise, an unpaired Wilcoxon test was performed. Where the average difference between two sample groups is cited, the error associated with the difference was calculated by adding the coefficients of variation from each data group, and the resulting sum was then multiplied by the difference.

### Variant nomenclature and accession

The variants described in this paper are done so in accordance with HGVS recommendations. Nucleotide numbering is based on reference GenBank sequence NM_000527.4. All variants described are available on ClinVar.

## Results

### *LDLR* 5’UTR variants

**Fig. 1:**
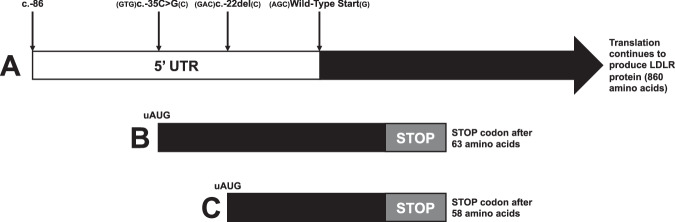
Schematic of 5’UTR and WT translated *LDLR* exon 1. The variants c.−35C > G and c.−22del are shown imposed on the 5’UTR **A**. Hypothetical translated products starting from the uATGs resulting from c.−35C > G and c.−22del are shown below as **B**, **C** respectively. c.−35**C** > G, c.−22del, and the wild type ATG have their respective Kozak consensus sequences shown in flanking brackets.

The 5’UTR analysis pipeline, utilising UTRannotator, was applied to 536 participants of the FH cohort in 100KGP. One 5’UTR variant, NM_000527.5(*LDLR*):c.−35C > G ((Accession: VCV003893685.1) (https://www.ncbi.nlm.nih.gov/clinvar/variation/3893685/)) was identified as potentially affecting the open reading frame of *LDLR* (Table 1) (Fig. 1). This variant is a single nucleotide variant (SNV) located 35 base pairs upstream of the 1st base of the protein-coding sequence (CDS) of *LDLR* and consists of a cytosine to guanine transversion. UTRannotator classified this variant as an upstream start (uAUG) gain variant with a moderate Kozak consensus, which is predicted to introduce a novel overlapping open reading frame (oORF) 63 amino acids in length. This variant was found in an FH proband with no Tier 1 or Tier 2 variants (https://re-docs.genomicsengland.co.uk/tiering). In gnomAD 4.1.0 one non-Finnish European out of 798,530 sequenced individuals was found to carry the variant, and no other carriers were identified in the 77,277 100KGP non-FH participants.

**Table 1:** Information of c.−35C > G variant with data derived from UTRannotator, also included is the c.−22del variant.

Chrom:Pos	Ref	Alt	DNA change	Variant type	Consequence	Distance to CDS	Distance to next in-frame STOP	Kozak Context	GnomAD (v4) AF
19:11089514	C	G	c.−35C > G	5’ UTR, uAUG gain	Creation of oORF	37	186	GTGATGC	0.0000007
19:11089527	C		c.−22del	5’ UTR, uAUG gain	Creation of oORF	24	173	GACATGC	0

*Chrom* Chromosome, *Pos* Position, *Ref* Reference allele, *Alt* Alternate allele, *CDS* Coding sequence, *AF* Allele frequency.

A literature search for other *LDLR* 5’UTR variants associated with FH found a single-nucleotide deletion at position −22, NM_000527.5(*LDLR*):c.−22del ((Accession: VCV000250962.1) (https://www.ncbi.nlm.nih.gov/clinvar/variation/250962/)), reported by Sözen et al. that is predicted to introduce a new ATG start codon and 58 amino acid long open reading frame out of frame with the wild-type CDS [14]. The variant was identified in a compound heterozygous patient presenting with cholesterol levels indicative of a homozygous familial hypercholesterolaemia (FH) phenotype (Total cholesterol (mmol/l): 20.12, LDL (mmol/l): 18.41, with xanthomas and CHD at age 8 years). Genetic analysis showed that the father carried only the c.−22del variant, while the mother had both *LDLR* c.762 G > T p.(Gln254His) and c.763 T > G p.(Cys255Gly) alterations on the same allele. The father, from whom the proband inherited the variant, had a phenotype consistent with heterozygous FH and total cholesterol of 8.83 mmol/L (HDL: 0.95 mmol/L, LDL: 6.73 mmol/L) [14]. The c.−22del variant was not found in gnomAD 4.1.0 or in any participant in the 100KGP.

### FH-associated 5’UTR variants reduce expression of the wild-type CDS

**Fig. 2:**
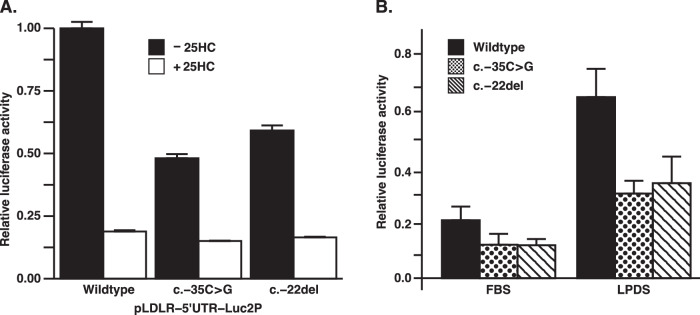
Measurement of luciferase reporter activity. On day 1 of growth, A549 cells in 96-well plates were transfected with 1 ng/well of phRL-CMV (expressing *Renilla* luciferase) plus 125 ng/well of pLDLR-UTR-Luc2P reporter plasmids containing either the wild-type *LDLR* 5’UTR or 5’UTR sequences with the indicated mutations. On day 2, cells were switched to cholesterol-depleting medium B in the absence or presence of 1 µg/ml 25-hydroxycholesterol (25HC). Cells were harvested and lysates analysed on day 3. Firefly luciferase activity was divided by *Renilla* luciferase activity. The results from three independent experiments were each normalised and then averaged. Error bars indicate SEM. **A** Relative firefly luciferase activity in DMEM/F12 supplemented with antibiotics (100 units per ml of penicillin and 100 µg per ml of streptomycin sulphate), 5% lipoprotein-deficient serum (LPDS), 0.5 µM lovastatin and 50 µM mevalonate (LPDS/Lov) and LPDS/Lov plus 25-hydroxycholesterol (LPDS/Lov + 25HC). **B** Relative firefly luciferase activity in all tested media; DMEM/F12 supplemented with antibiotics and 10% FBS (FBS), DMEM/F12 supplemented with antibiotics, 5% lipoprotein-deficient serum (LPDS), LPDS/Lov, and LDPS/Lov +25HC.

If the novel AUG codons introduced in the 5’UTR were used preferentially for the initiation of translation, the variants would be expected to reduce the expression of the wild-type *LDLR* open reading frame. To test this hypothesis, we constructed plasmids in which a reporter gene encoding firefly luciferase (FLuc2P) was preceded by the promoter (bases −428 to −93) plus 5’UTR (bases −92 to +4) of the human *LDLR* gene, such that the entire Kozak sequence (AGCATGG) at the luciferase translation initiation site corresponded to that of the *LDLR*. Following transfection, cells were grown in cholesterol-depleting medium containing lipoprotein-deficient serum (LPDS) and lovastatin to promote maximal expression through the promoter’s SREBP response element [25]. A low concentration of mevalonate was supplemented to ensure survival of the cells in the presence of lovastatin. As a control, some cells received SREBP-suppressing 25-hydroxycholesterol to confirm normal regulation of the reporters. As shown in Fig. 2A (black bars), expression of firefly luciferase was reduced by 59.4% ± 4.5% (Mean ± SEM, *p* < 0.0001) following the introduction of the c.−35C > G variant. For the plasmid with the c.−22del variant, expression was reduced by 46.7% ± 3.4% (*p* < 0.002). In Fig. 2B similar results can be seen for cells grown purely with in standard growth medium containing FBS or for cells grown with LPDS (without lovastatin and mevalonate). Compared to wild-type, expression of the c.−35C > G variant expression was reduced by 42.3% ± 4.9% (FBS, *p* = 0.01) and 53.3% ± 10.7% (LPDS, *p* < 0.0001), respectively. In cells transfected with the c.−22del variant, expression was reduced by 43.1% ± 3.8% (FBS, *p* = 0.01) and 47.5% ± 13.5% (LPDS, *p* = 0.004).

**Fig. 3:**
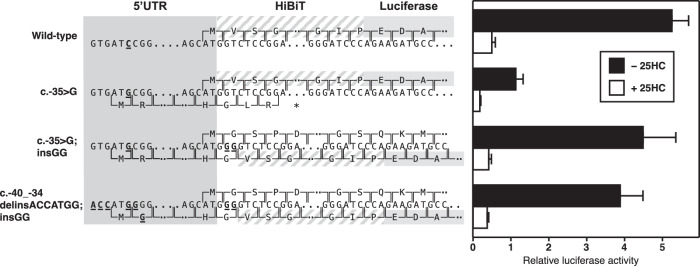
Measurement of luciferase and HiBiT reporter activity. A549 cells were grown as in Fig. 2 and transfected with 10 ng/well of pCATlac (expressing β-galactosidase) plus 125 ng/well of pLDLR-UTR-HiBiT-Luc2P reporter plasmids. On day 2, cells were switched to cholesterol-depleting medium B in the absence or presence of 1 µg/ml 25-hydroxycholesterol (25HC). Enzyme activities were analysed on day 3. NanoLuc (indicating HiBiT expression) was corrected for β-galactosidase. Error bars indicate SD (n = 3). Sequences that differ between the four reporters are highlighted in red. Plasmid (b) differs from wild-type plasmid (a) by a c.−35C > G mutation in the *LDLR* 5’UTR; the mutation introduces an upstream AUG that is out of frame with the HiBiT-Luc2P CDS. Plasmid (c) differs from plasmid (b) by the insertion of two G nucleotides following the LDLR Kozak sequence, which brings HiBiT-Luc2P in frame with the upstream AUG. Plasmid (d) differs from plasmid (c) in that sequences surrounding the upstream AUG were modified to match the core Kozak consensus sequence.

To test whether the novel ATG sequence at position −35 of the UTR is in fact used for the initiation of translation, we constructed another set of plasmids in which the *LDLR* promoter and 5’UTR preceded a firefly luciferase gene with an N-terminal HiBiT tag. The HiBiT peptide can be detected with a luminescence assay through complementation of a split NanoLuc luciferase system [23]. Confirming the results in Fig. 2, HiBiT expression in cholesterol-depleting medium was high when the HiBiT-FLuc2P fusion protein was cloned in frame with the *LDLR* translation initiation codon (Fig. 3, “Wild-type” construct) but reduced by about 79% following introduction of a novel ATG at position −35 of the 5’UTR (construct “c.−35C > G”). Expression of the reporter was restored, however, when the HiBiT-FLuc2P CDS was brought in frame with the novel ATG (construct “c.−35C > G; insGG”), measurements of FLuc2P activity in the same extracts mirrored those obtained for the HiBiT tag (data not shown).

We were initially surprised by the high expression of the reporter when cloned in frame with the novel AUG because the surrounding sequence is a very poor match for the Kozak consensus sequence [26]. To further explore this observation, we generated another reporter plasmid in which the HiBiT-FLuc2P CDS was again in frame with the AUG at −35 but the nucleotides surrounding the novel initiation codon were changed to match the core Kozak sequence (construct “c.−40_−34 delinsACCATGG; insGG”). The expression of this reporter was comparable to that of the reference construct. Taken together, the results in Figs. 2 and 3 show that the c.−35C > G variant leads to preferential initiation of translation from the novel AUG, leading to a significant reduction of protein synthesis from the usual LDLR translation start site.

### Variant classification with The American College of Medical Genetics and Genomics (ACMG) guidelines

To assess the pathogenicity of the c.−35C > G and c.−22del *LDLR* variants, combined criteria from the ACMG guidelines adapted by Chora et al., specific to FH, and Ellingford et al., specific to non-coding variants, were used [27, 28]. The c.−22del variant was not present in GnomAD v4.1.0, and c.−35C > G had a PopMax MAF ≤ 0.0002 (0.02%), therefore both meet the PM2 moderate pathogenic criteria as they are sufficiently rare in the population (PM2, Chora et al.). The variants were found in patients who have been diagnosed with FH, therefore PP4 supporting pathogenic criteria for a variant present in a patient with FH (Chora et al.) was applied. *LDLR* haploinsufficiency is a cause of FH and the variants have the same predicted impact as previously identified pathogenic variants, which cause *LDLR* haploinsufficiency [29, 30]. Translation from each variant’s resultant uAUG would create an overlapping open reading frame (oORF) that overlaps the coding sequence out-of-frame with the canonical start site (PM5, Ellingford et al.). Luciferase functional assays demonstrated a 59.4% ± 4.5% (Mean ± SEM) reduction in activity compared with wild type, which is sufficient to apply the PS3_Supporting (Chora et al.) criterion for c.−35C > G. For the c.−22del variant a < 50% reduction in activity was observed (46.7% ± 3.4% (Mean ± SEM)) therefore not sufficient for PS3_Supporting criteria (Chora et al.). Following this, for the c.−22del variant, we are unable to apply PP1 as currently the segregation data is only available for one family (as per Chora et al. guidelines) [14]. Additionally, we are unable to apply PM3 as the other two variants ((NM_000527.5(*LDLR*):c.762 G > T, NM_000527.5(*LDLR*):c.763 T > G)) the proband inherited in *trans* both have conflicting classifications of pathogenicity and therefore are classified as VUSs at the time of writing (Chora et al.) [14]. Altogether there is sufficient evidence for c.−35C > G to be classified as Likely Pathogenic, while c.−22del met criteria of a VUS.

## Discussion

Here we demonstrate a novel molecular mechanism for non-coding variation in *LDLR* leading to FH. Luciferase assays comparing wild-type *LDLR* 5’UTR to the c.−35C > G and c.−22del variants confirm a significant reduction in *LDLR* promoter activity, of 59.4% and 46.7% activity, respectively. These variants are thus likely to lead to an impaired expression of LDL receptors, which would explain the insufficient clearance of LDL-C from the blood of the affected probands.

Both sequence changes lead to the introduction of novel uAUG codons that are out of frame with the wild-type translation start codon. In humans, translation is initiated when the small ribosomal subunit encounters the first AUG codon, which let us to hypothesise that the c.−35C > G and c.−22del variants reduce expression due to the competition of the novel and normal start sites for the ribosome. This hypothesis was borne out by experiments where a reporter CDS was cloned in frame with the variant uAUGs (Fig. 3). Although the sequence surrounding the uAUG introduced at position −35 is a poor match to the consensus initiation site [26], the inclusion of an ideal Kozak sequence around this uAUG (construct “c.−40_34 delinsACCATGG; insGG”) did not enhance translational efficiency of the in-frame reporter, suggesting that the presence of a uAUG alone is sufficient to disrupt LDLR protein production.

The short peptides that are predicted to be produced from the two uAUG variant have an unknown function at present, and it is unclear whether they have any biological effect.

Our findings underscore the importance of considering non-coding variants in genetic diagnostics for FH, as they can significantly impact gene expression even when the coding sequence appears intact. However, compared to other pathogenic variants in the *LDLR* promoter, these uAUG creating variants have a somewhat stronger effect. SP1 binding site variants were shown to alter promoter activity (% of wildtype) by 6−26%, SREBP2 binding site variants 11−40%, and SREBP1 binding site variants 10% [31–34]. Therefore, identification of a likely pathogenic uAUG creating variant can provide the patient with a genetic diagnosis, which can then be used for cascade testing and to personalise the treatment.

The use of UTRannotator in a large sequencing dataset was particularly valuable in identifying the c.−35C > G variant as a uAUG gain variant and predicting its impact on translation [21]. In addition to potential implementation in current FH genetic testing of *LDLR*, this tool can be further employed to investigate other important FH-related genes. For example, the *PCSK9* mRNA encodes a uORF with the uAUG at Chr1-55039566-55039568. Loss-of-uORF variants are thought to be associated with enhanced translation [35–37], which in the case of PCSK9 would be expected to cause increased turnover of the LDLR protein and might thus impair the clearance of LDL. Though our study did not find any such uAUG loss variants in the 100KGP cohort, 5 SNVs are listed in GnomAD v4.1.0 which would cause a uAUG loss (all described SNVs have MAF of <0.0001). Whether these SNVs are associated with hypercholesterolaemia has not yet been reported.

One limitation of our study is the inability to access lipid profile data for the 100KGP FH cohort due to Genomics England confidentiality constraints. This restricts our capacity to fully correlate the functional impacts of identified variants with phenotypic data across the entire cohort but all patients within the 100KGP FH cohort are confirmed to have been diagnosed according to the Simon Broome criteria. Family co-segregation of the c.−35 variant and collection of patients cells to perform western blotting to confirm the expression defect were not possible due to the loss of contact with the study participant.

The implications of this research are twofold. First, it highlights the need for broader screening of non-coding regions in FH diagnostics, as variants like c.−35C > G and c.−22del may contribute to the disease in patients lacking known pathogenic variants in coding regions. Second, it provides a potential molecular mechanism by which non-coding variants can contribute to FH, specifically through the creation of uORFs that interfere with normal translation. Future studies should focus on expanding the catalogue of 5’UTR variants in FH patients and investigating their functional impact using in vitro and in vivo models.

## Supplementary information

Supplementary Methods

## Data Availability

Genetic and phenotypic data for the 100KGP study participants are available through the Genomics England Research Environment via the application at https://www.genomicsengland.co.uk/research/academic/join-research-network.
