# Supplementary material for: Novel start codon variant in the 5’UTR of LDLR associated with familial hypercholesterolaemia
Source: Eur J Hum Genet. 2025 Jul 24;34(4):522–7. doi: 10.1038/s41431-025-01893-y (PMC13046971; doi:10.1038/s41431-025-01893-y)
Supplement: Supplementary file 1 — Supplementary Methods [file 41431_2025_1893_MOESM1_ESM.docx]

**SUPPLEMENTARY MATERIAL**

**Novel start codon variant in the 5’UTR of *LDLR* associated with Familial Hypercholesterolaemia.**

Martin Bird^1^, Chris Jyun-Peng Tung^2^, Alan Pittman^1^, Elijah R Behr^1^, Axel Nohturfft^2^, and Marta Futema^1,3^

^1^Cardiovascular and Genomics Research Institute, School of Health & Medical Sciences, City St George's, University of London, London, UK

^2^Neuroscience and Cell Biology Research Institute, School of Health & Medical Sciences, City St George's, University of London, London, UK

^3^Institute of Cardiovascular Science, Faculty of Population Health, University College London, London, UK

Corresponding author: [mbird@sgul.ac.uk](mailto:mbird@sgul.ac.uk) and [mfutema@sgul.ac.uk](mailto:mfutema@sgul.ac.uk)

**SUPPLEMENTARY METHODS**

**S1 Reagents**

Reagents were sourced as follows: DMEM/F12 medium (Gibco brand) and oligonucleotides from Life Technologies (Paisley, UK); antibiotics, DL-mevalonolactone (Sigma brand, M4667), fetal bovine serum (Sigma brand), ortho-nitrophenyl-β-galactoside (ONPG) and single-stranded oligonucleotides from Merck (Darmstadt, Germany); 25-hydroxycholesterol from Cambridge Bioscience (Bar Hill, UK); fetal, bovine lipoprotein-deficient serum (LPDS; AlphaDiagnostics brand) and lovastatin from CliniSciences Ltd (Slough, UK); and double-stranded oligonucleotides from Life Technologies. To prepare a stock solution of mevalonic acid, mevalonolactone was dissolved in water, hydrolysed by adding an equal volume of 1N KOH, incubated at 37˚C for 1 hour and neutralized with 1M HCl; the final concentrations was then adjusted to 50 mM mevalonate and 1x phosphate-buffered saline.

**S2 Oligonucleotides replacing BmgBI-NcoI fragment of pLDLR-Luc2p corresponding to -97 to -2**

**_Oligos (wild-type):_**

_5’- GTGGGCCCCGAGTGCAATCGCGGGAAGCCAGGGTTTCCAGCTAGGACACAGCAGGTCGTGATCCGGGTCGGGACACTGCCTGGCAGAGGCTGCGAG -3’_

_3’- CACCCGGGGCTCACGTTAGCGCCCTTCGGTCCCAAAGGTCGATCCTGTGTCGTCCAGCACTAGGCCCAGCCCTGTGACGGACCGTCTCCGACGCTCGTAC -5’_

_Lower strand 5’to3’:_

_5’-CATGCTCGCAGCCTCTGCCAGGCAGTGTCCCGACCCGGATCACGACCTGCTGTGTCCTAGCTGGAAACCCTGGCTTCCCGCGATTGCACTCGGGGCCCAC-3’_

**_Oligos (c.-35C>G):_**

_5’- GTGGGCCCCGAGTGCAATCGCGGGAAGCCAGGGTTTCCAGCTAGGACACAGCAGGTCGTGATGCGGGTCGGGACACTGCCTGGCAGAGGCTGCGAG -3’_

_3’- CACCCGGGGCTCACGTTAGCGCCCTTCGGTCCCAAAGGTCGATCCTGTGTCGTCCAGCACTACGCCCAGCCCTGTGACGGACCGTCTCCGACGCTCGTAC -5’_

_Lower strand 5’to3’:_

_5’-CATGCTCGCAGCCTCTGCCAGGCAGTGTCCCGACCCGCATCACGACCTGCTGTGTCCTAGCTGGAAACCCTGGCTTCCCGCGATTGCACTCGGGGCCCAC-3’_

**_Oligos (c.-22del):_**

_5’- GTGGGCCCCGAGTGCAATCGCGGGAAGCCAGGGTTTCCAGCTAGGACACAGCAGGTCGTGATCCGGGTCGGGACATGCCTGGCAGAGGCTGCGAG -3’_

_3’- CACCCGGGGCTCACGTTAGCGCCCTTCGGTCCCAAAGGTCGATCCTGTGTCGTCCAGCACTAGGCCCAGCCCTGTACGGACCGTCTCCGACGCTCGTAC -5’_

_Lower strand 5’to3’:_

_5’-CATGCTCGCAGCCTCTGCCAGGCATGTCCCGACCCGGATCACGACCTGCTGTGTCCTAGCTGGAAACCCTGGCTTCCCGCGATTGCACTCGGGGCCCAC-3’_
